# Human Gene Coexpression Landscape: Confident Network Derived from Tissue Transcriptomic Profiles

**DOI:** 10.1371/journal.pone.0003911

**Published:** 2008-12-15

**Authors:** Carlos Prieto, Alberto Risueño, Celia Fontanillo, Javier De Las Rivas

**Affiliations:** Bioinformatics and Functional Genomics Research Group, Cancer Research Center (CIC-IBMCC, CSIC/USAL), Salamanca, Spain; University of Toronto, Canada

## Abstract

**Background:**

Analysis of gene expression data using genome-wide microarrays is a technique often used in genomic studies to find coexpression patterns and locate groups of co-transcribed genes. However, most studies done at global “omic” scale are not focused on human samples and when they correspond to human very often include heterogeneous datasets, mixing normal with disease-altered samples. Moreover, the technical noise present in genome-wide expression microarrays is another well reported problem that many times is not addressed with robust statistical methods, and the estimation of errors in the data is not provided.

**Methodology/Principal Findings:**

Human genome-wide expression data from a controlled set of normal-healthy tissues is used to build a confident human gene coexpression network avoiding both pathological and technical noise. To achieve this we describe a new method that combines several statistical and computational strategies: robust normalization and expression signal calculation; correlation coefficients obtained by parametric and non-parametric methods; random cross-validations; and estimation of the statistical accuracy and coverage of the data. All these methods provide a series of coexpression datasets where the level of error is measured and can be tuned. To define the errors, the rates of true positives are calculated by assignment to biological pathways. The results provide a confident human gene coexpression network that includes 3327 gene-nodes and 15841 coexpression-links and a comparative analysis shows good improvement over previously published datasets. Further functional analysis of a subset core network, validated by two independent methods, shows coherent biological modules that share common transcription factors. The network reveals a map of coexpression clusters organized in well defined functional constellations. Two major regions in this network correspond to genes involved in nuclear and mitochondrial metabolism and investigations on their functional assignment indicate that more than 60% are house-keeping and essential genes. The network displays new non-described gene associations and it allows the placement in a functional context of some unknown non-assigned genes based on their interactions with known gene families.

**Conclusions/Significance:**

The identification of stable and reliable human gene to gene coexpression networks is essential to unravel the interactions and functional correlations between human genes at an omic scale. This work contributes to this aim, and we are making available for the scientific community the validated human gene coexpression networks obtained, to allow further analyses on the network or on some specific gene associations.

The data are available free online at http://bioinfow.dep.usal.es/coexpression/.

## Introduction

Exploration and analysis of gene expression data using genome-wide microarrays is a technique often used in genomic studies to find coexpression patterns and locate groups of co-transcribed genes. This kind of studies has been used in model organisms, like yeast [1], to discover gene functions, to define biological processes and to find related transcription factors and their products. The main features of expression patterns that give a wide utility in bioinformatic studies are: the functional information associated [2], the high conservation of gene coexpression groups along evolution [3] and the high correlation of these groups with biomolecular pathways or reactions [4]. All these features leverage genome-wide expression profiling, and convert this topic in a hot research area.

Despite the described interest, coexpression studies done at global “omic” scale are not focused in many cases on human samples [5], and, when they correspond to human, very often they include heterogeneous datasets, mixing “normal” samples with “disease altered” samples from patients suffering from some kind of pathological state. This is the case, for example, in several human gene expression large studies [2], [6]. The inclusion of many disease datasets (mainly from cancer) in such meta-analyses may introduce strong bias and produce a lot of biological noise in the results. In fact, it is well known that cancer cells have altered genomes. Therefore, these kind of studies cannot be used to clarify how a normal-healthy human cellular system works, and they cannot be used to draw a reliable map of the human gene coexpression landscape.

The technical noise in the genome-wide expression microarray studies is another well reported problem that can not be ignored when gene coexpression studies at “omic” scale are undertaken. Considering all these problems and knowing the interest of having a reliable normal human gene coexpression network, we have undertaken this task selecting human genome-wide expression microarrays from a controlled set of different normal tissues to build a confident human transcriptomic network using several statistical and computational methods. These methods (which include robust data normalization and signal calculation, combined parametric and non-parametric correlation and random cross-validation) help to avoid both biological and technical noise and provide a human gene coexpression network that shows good accuracy and coverage. Moreover, the network reveals well defined biological functions and pathways that map to specific coexpression clusters.

## Results and Discussion

### Genome-wide expression profiles from a broad set of human samples

**Figure 1:**
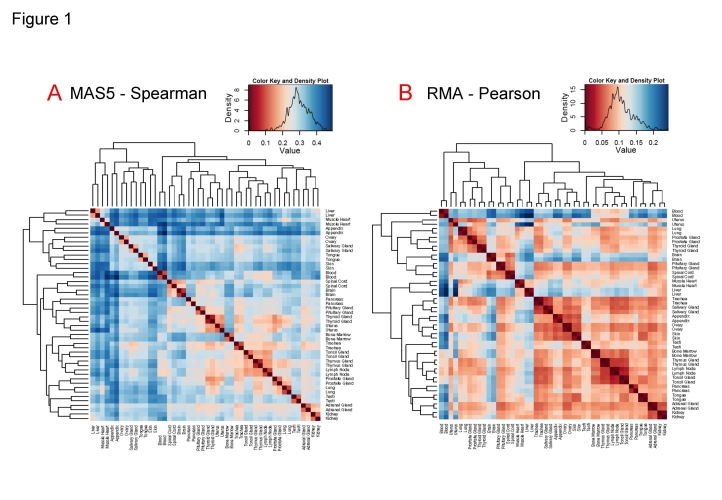
Clustering of human tissue expression profiles. Heatmaps and clustering of the 48 human genome-wide expression microarray samples from 24 different tissues and organs analyzed by two different methods: (A) MAS5-Spearman: MAS5 for signal calculation and Spearman for distance calculation based on the sample expression profiles; and (B) RMA-Pearson: RMA for signal calculation and Pearson for distance calculation based on the sample expression profiles. A color bar with scales for each heatmap is included, indicating that dark-red corresponds to minimum distance and dark-blue to maximum distance. The color distributions observed in the heatmaps are also included inside the bars.

An expression matrix was calculated for a dataset of human genome-wide microarrays hybridized with mRNA samples coming from different human tissues, glands and organs from healthy normal individuals. As indicated in **Materials and Methods** the dataset included two biological replicates of samples from 24 parts of the body: *adrenal gland*, *appendix*, *blood*, *bone marrow*, *brain*, *kidney*, *liver*, *lung*, *lymph node*, *muscle heart*, *ovary*, *pancreas*, *pituitary gland*, *prostate gland*, *salivary gland*, *skin*, *spinal cord*, *testis*, *thymus gland*, *thyroid gland*, *tongue*, *tonsil gland*, *trachea* and *uterus*. **Figure 1** presents the heatmaps and clustering of the 48 samples analyzed by two different methods following the strategy and steps described in **Methods**: **(1^st^)** “MAS5-Spearman” method, that applies MAS5 algorithm for signal calculation and Spearman correlation coefficient (**r**) for distance calculation (based on the sample expression profiles and displayed in the heatmap as **1−r**); **(2^nd^)** “RMA-Pearson” method, that applies RMA algorithm for signal calculation and Pearson correlation coefficient (**r**) for distance calculation (also based on the sample expression profiles and displayed as **1−r**). We use “Spearman with MAS5” and “Pearson with RMA” because it has been shown that the inclusion of at least one non-parametric step based on ranks in the analyses of microarray data offers statistically more robust and more accurate estimation of expression values [7] and expression correlations [8]. The two methods proposed provide such non-parametric transformation (i.e. change to ranks), because Spearman is a rank correlation coefficient and RMA includes a quantile normalization.

The heatmaps **(** **Figures 1A and 1B** **)** show a clear and coherent clustering of each pair of biological replicates. A color bar with scales for each heatmap is included in the figure, indicating that **dark-red** corresponds to minimum distance (i.e. maximum correlation) and **dark-blue** to maximum distance (i.e. minimum correlation). White color corresponds to medium values and the distributions inside the color bars show that the two methods are similar but not identical: MAS5-Spearman provides larger distances between samples (more blue values in the heatmap) than RMA-Pearson (more red values in the heatmap). The similarity and proximity of the replicates is closer in the case of the second method, but in both cases there is not confusion or separation of any pair of replicates. By contrast to this clear clustering, the ordering and clustering of the different tissues, glands and organs is not fixed in the heatmaps, changing quite a lot from **1A** to **1B**. This observation was confirmed by bootstrap analysis done with *pvclust* [9] which allows the assessment of the uncertainty in hierarchical clusters (see **Methods**). The results of *pvclust* showed that the biological “replicate pairs” gave in all cases stable groups with optimum probability values (AU and BP = 100%). However, within the tissues and organs only two stable groups were found with both methods: the group that includes *lymph node*, *thymus gland* and *tonsil gland* (that gave a AU value of 0.98); and the group that includes *kidney* and *adrenal gland* (with AU value 0.97). These groups have clear biological meaning since they correspond to physiologically and functionally related organs (i.e. *lymph node*, *thymus* and *tonsil* are related to the lymphatic and immune systems). Thus the functional relationship between samples is captured by the gene expression profiles. However, all the other tree branches produced low AU values, therefore the overall sample clustering observed in the heatmaps indicates a lack of well defined and stable groups. In conclusion, these results show neat separation of most of the sample expression profiles, which is an adequate condition for the exploration of a global broad human gene expression landscape.

In order to consider if these observations are reliable enough, we explored the data changing some conditions following another two different strategies (data not shown). **First** strategy, the same analyses with 48 microarrays were done again twice: one not using the total number of genes (i.e. 22 283 gene probesets) but only the 25% of the genes that showed the largest variance; and another using only the 25% of the genes that showed the highest signal. In both cases, the heatmap and trees obtained were very similar to the ones presented in **Figure 1**, and the bootstrap gave similar results. **Second** strategy, we included in the data set two new groups of microarrays corresponding to samples from specific organs: 16 microarrays from different parts of the brain and 10 microarrays from different hematologic cell types. In this case (data not shown) the analyses provided larger trees, where two main clusters were segregated from other branches: one corresponding to brain related samples (i.e. nervous system) including the two whole-brain samples; and another cluster corresponding to the hematologic related samples including the two whole-blood samples. These results indicate again that any functional relation between samples is well captured by the global gene expression profiles, and provide validity to the genome-wide expression profiles of human normal tissues obtained, allowing us to proceed to the next step of the study.

### From sample expression profiles to gene expression signatures

The main data presented so far correspond to the analysis of the genome-wide expression profiles of samples from different human normal tissues, organs or glands. These genome-wide “sample profiles” are numerical vectors including the expression values of each one of the gene probesets present in the microarray (i.e. each one of the detectable genes of the human genome). As shown above, the “sample profiles” can resemble the physiological relationships expected between the samples (tissues, glands and organs). However, in order to achieve a mapping of the human gene coexpression landscape, we needed to move from the analysis of the “sample expression profiles” based on the genes, to the analysis of each “gene expression signature” based on the sample set.

It is difficult to achieve a proper gene coexpression study due to several obstacles that have to be taken in consideration: **(i)** the technical noise present in the microarrays at genomic scale [10], despite the fact that the *Affymetrix* high density oligonucleotide genechips have been reported quite reliable and reproducible [11], [12]; **(ii)** the small number of samples used to define each gene expression signature (specially in comparison to the large number of genes); **(iii)** the strong heterogeneity of the data sets frequently studied, that include in many cases samples from pathological or altered states [2], [13] which are not adequate samples to find “normal” gene expression behavior.

The approach and strategies taken in this study to solve or minimize these problems were the following: **(a)** careful selection of expression samples from different parts of the human body (tissues, whole glands and whole organs) from normal healthy individuals; **(b)** calculation of expression signals and correlations using two different independent methods: MAS5-Spearman, RMA-Pearson; **(c)** use of a robust random cross-validation strategy to find the most stable correlation pairs and distinguish the consistent biological-signal from the noise-signal; **(d)** statistical estimation of the accuracy and the coverage for each coexpression dataset obtained. All the details and description of these strategies are presented in **Materials and Methods**. The results associated with them have been partially described above and are explained in the following paragraphs.

### Gene pairs coexpression analyzed with cross-validated correlations

**Figure 2:**
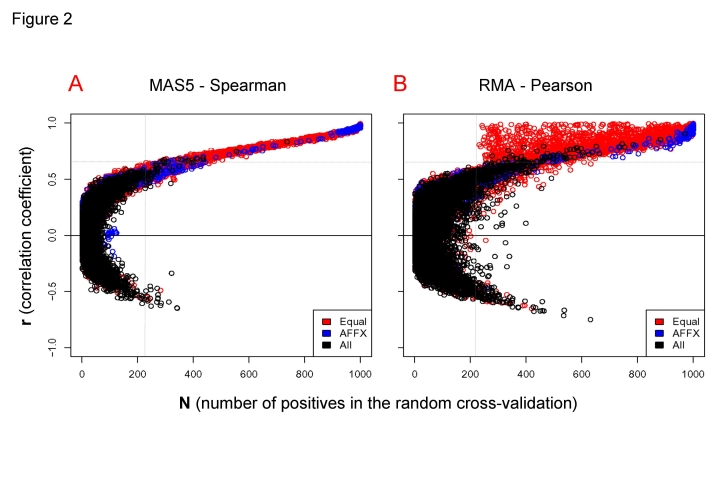
Plot of r and N coefficients calculated for each gene coexpression pair. rN-plots that represent the correlation coefficient (from 0 to 1) versus the cross-validation coefficient (from 0 to 1000) of each gene pair by two different methods: (A) MAS5-Spearman and (B) RMA-Pearson. The cross-validation is considered positive for a given gene pair when it gives r>|0.7| in each sampling. As indicated in Methods 1000 samplings are run for each gene-probeset pair. The gene probeset pairs that correspond to the same gene are drawn as red circles. The probeset pairs of *Affymetrix* controls are drawn as blue circles. A random selection of 10,000 coexpressed gene probeset pairs are drawn as black circles. Two dotted lines are drawn to indicate an approximate threshold that can be considered the border of noisy data. These lines are drawn just to show the minimal r and N values bellow which the coexpressed gene pairs are mainly noise; therefore the coexpression signal appears mostly at r>0.65 and N>220.

The complete expression data matrix analyzed had, as indicated, 48 samples (24 duplicates) and 22,283 gene probesets (which correspond to 13,068 distinct known human genes according to *Affymetrix* annotation). Therefore the global pair-wise gene coexpression matrix including all possible pairs had 248,254,903 data points and was calculated twice, once for each independent method used (MAS5-Spearman and RMA-Pearson). These huge data matrices have many pairs that are false coexpression pairs and to detect those positive gene pairs that had stable and significant correlation we use cross-validation. The results corresponding to the gene pairs correlation obtained with the cross-validation method (described in **Methods**) are presented in **Figure 2**, that shows what we called “**rN-plots**”. The rN-plots are graphics representing: **r** at *y* axis, that is, for each gene probeset pair, the “correlation coefficient” of their expression signatures along the complete dataset of 48 samples, calculated as Spearman or Pearson distance (for MAS5 or RMA data, respectively) (with values from 0 to 1 for positive correlations and from 0 to −1 for negative correlations); **N** at *x* axis, that is the “cross-validation coefficient” defined as the number of times that a given gene pair has a significant correlation (i.e. r≥|0.70|) out of the 1000 times random selection (as explained in **Methods**). This graphical analysis presents the positive and negative correlations well segregated and it allows to identify those gene pairs that have a significant “cross-validated correlation”, discriminated from those false gene-pairs that have low **r** or low **N** values. Such false gene-pairs do not correlate in a stable and consistent way, being undistinguishable from noise.

To demonstrate how the **rN-plots** represent stable and consistent correlations, we selected in the case of the **red** circles or dots only the gene probeset pairs that correspond to probesets assigned to “the same gene”. For example, pairs between the 4 probesets that correspond to gene ALDOB, *fructose bisphosphate aldolase B* (204704_s_at, 204705_x_at, 211357_s_at, and 217238_s_at in microarray HGU133A); or pairs between the 3 probesets that correspond to gene CDK10, *cell division protein kinase 10* (203468_at, 203469_s_at and 210622_x_at in HGU133A). When correlation is found between these kind of “common gene probesets” they are drawn as **red** circles in **Figure 2**. The analysis indicates that the red circles accumulate at high **r** correlations and high **N** values. This is the result that should be expected considering that these groups of probesets are measuring the same gene; and, despite the fact that this is not always true, it is a good way to evaluate the meaning of the **rN-plot**. A more stringent evaluation was to find out the correlation between probesets that correspond to “control RNAs” that are added in each microarray assay in the hybridization process. Such controls, named with prefix AFFX in the chip, are spike controls (i.e. series of mRNAs added during hybridization protocol that correspond to different concentrations of non-human genes like AFFX-BIO) and human house-keeping controls (like AFFX-HUMGAPDH). These controls should have strong correlation since they have been added to the microarrays in known concentrations. We draw such correlations in the **rN-plots** as **blue** circles (**Fig. 2**); and it could be seen that the distribution of these true positive gene correlated pairs was very much accumulated at high **N** values and high **r** correlations. This observation again shows that the **rN-plots** are very useful and valuable to separate noisy false correlations from stable true correlations.

The differences observed between **Fig. 2A and 2B** are due to the differences in the methods and to the characteristics of the cross-validation (described in **Methods**). Some **red** circles with high-**r** and low-**N** appear only in the RMA-Pearson method because the correlations derived from this method give in some instances high correlation values to gene pairs that are correlated just in only one tissue (shown in **Fig. 2B**). The cross-validation values of these gene pairs are low because they only appear when such one tissue samples are selected. The probability to select one sample pair out of 24 is: 1−(23/24)^6^ = 0.225; and this is why the red circles with high-**r** and low-**N** only appear for values **N**>225. By contrast, the MAS5-Spearman method does not find any **red** circle in the high-**r** and low-**N** region, because Spearman is a “rank correlation coefficient” which does not produce high correlation values for gene pairs that correlated in only one tissue (just once out of 6). The **r** value obtained with the Spearman method is proportional to the number of tissues or samples that co-express and so it is quite proportional to **N**.

### Data filtering to clear genes with low information content

The calculations and analysis presented in **Figure 2**, were done without using any previous filter of gene probesets. No filtering means using the complete gene expression matrices with all the human gene probesets present in the microarrays. It is known that in most samples and conditions genome-wide microarrays include a large proportion of the genes that are not expressed and therefore they give signal close to the background or noise. This situation is not very likely to occur all along the complete sample set of 24 different tissues and organs studied here. However, out of the 22,283 gene probesets some may have no significant change, and therefore, it is important to find out the possible presence and effect of these “non-changing genes” (that we also called “flat-genes”) [14]. The most adequate filter to be used in most of the expression analyses is a variance-filtering between samples (i. e. between-array variability), because this approach filters out elements of low information content within the sample set and covers the complete signal range (from low to high expression), therefore, it does not bias the data by signal intensity or signal ratios [14], [15]. However some genes with high signal may be significant despite showing relative low variance, and for these reasons it is better to apply combined filters that explore the variance, but also consider the intensity of the probes [15].

As described in **Methods** we use a combined filter based on between-sample variability and gene minimal signal, that is designed to get rid of genes with low information content. The use of this filter with the 48 microarrays sample set gave different results for the data expression matrix obtained with RMA method and the expression matrix obtained with MAS5 method. In the first case the filter leaves out 6,893 gene probesets (leaving 69.06%) and in the second 3,682 (leaving 83.48%) from 22,283 total gene probesets. The difference in these numbers shows that these two methods do not provide an equal calculation of expression signal and variance and therefore, as explained bellow, both methods can be considered complementary.

### Analysis of accuracy and coverage along gene coexpression data

Using the filtered data sets we follow a more thorough analysis of the coexpression distributions with respect to the parameters **r** and **N**. In the rN-plots (**Fig. 2**) two dotted lines were drawn to indicate an approximate threshold for coexpressed gene pairs that could be considered the border of noisy data. These lines are tentatively drawn just to show the minimal **r** and **N** values bellow which the coexpression pairs are mainly noise; therefore, the coexpression signal appears mostly at **r**>0.65 and **N**>220. However, this estimation is not robust enough and a proper calculation of the statistical “accuracy” and “coverage” along all the gene coexpression data matrices was done. The details about the calculation of these parameters are described in **Materials and Methods**. KEGG pathway database was used to estimate the true positives. After these calculations, for all data presented (i.e. all next **Figures**) the nodes correspond to genes and not any more to “gene probesets” from the microarrays. This change was done taking the correspondence of the probesets to the specific genes according to the *Affymetrix* annotation files for HG-U133A from 31.May.2007 (that can be found in URL: http://www.affymetrix.com/support/technical/byproduct.affx?producthgu133). In this conversion all probesets of the microarray were used. Previously, we calculated the coexpression values for each gene pair considering each probeset independently. When multiple probesets map to one gene, we merged the multiple probesets to the corresponding gene and we only take the gene coexpression pairs with maximum values of correlation (**r**) and cross-validation (**N**) in which its probesets participate.

**Figure 3:**
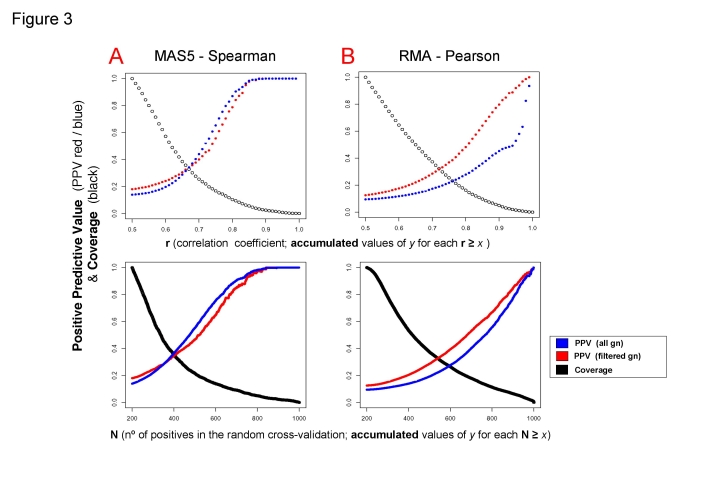
Accuracy and coverage of the coexpression data. Accuracy measured as Positive Predictive Value PPV (for all genes in blue and filtered genes in red) and coverage as True Positive Rate TPR (in black) computed for each coexpression dataset obtained at a given correlation coefficient r (top figures) or at a given number of cross-validations N (bottom figures) for both methods: (A) MAS5-Spearman and (B) RMA-Pearson. The accuracy and coverage (in *y* axis) correspond to accumulated values for each r≥*x* or for each N≥*x*.

In **Figure 3** the positive predictive value (PPV) was computed for each coexpression data set obtained at a given correlation factor **r** (**Fig. 3** top graphs) or at a given the number of cross-validations **N** (**Fig. 3** bottom graphs). The change or evolution of the accumulated PPV is drawn as a curve (solid **red** and **blue** circles) for both methods (**Fig. 3A**: MAS5-Spearman; **B**: RMA-Pearson). The graphs show that the rate of true positives increases with higher expression correlation and with higher number of cross-validation. The increase is more significant for the MAS5-Spearman method that achieves PPV about 80% for **r**≥0.8 and for **N**≥700. However, RMA-Pearson provides higher coverage since the amount of positive gene coexpression pairs annotated to common KEGGs for **r** and **N** values is quite different in both methods (larger for RMA-Pearson). The results for the coverage calculated for each method are shown by the curves in black in **Figure 3** (**black** circles), presenting the amount of gene pairs annotated to common KEGGs that remain at each **r**≥*x* or **N**≥*x*. This is calculated considering as “total amount of positive pairs” (value 1.0 at the beginning of the curve, 100%): the number of gene coexpressing pairs annotated to common KEGGs at **r**≥0.5 and **N**≥200. This coverage parameter indicates, as it should be expected, that the number of gene coexpressing pairs decreases when the conditions (**r** and **N**) are more stringent. The decrease is steeper for the MAS5-Spearman method since for **r**≥0.75 it retains about 16.7% of the positive data points, but RMA-Pearson retains 25.4%. Equally for **N**≥600 the MAS5-Spearman method retains 13.9% of the positive data points and RMA-Pearson retains 26.4%. The total amount of positive pairs, which corresponds to value 100% at the beginning of the curve, was: 15,657 for RMA-Pearson and only 2,198 for MAS5-Spearman. These numbers seem small but they only correspond to the “positive pairs”, and so, if we take the total number of gene probeset coexpression pairs of the study (i.e. not including only the genes annotated to KEGGs but the complete coexpression data sets) the figures are much larger: 1,340,472 for RMA-Pearson and 180,305 for MAS5-Spearman. These results also indicate that the coverage is larger with the RMA-Pearson method.

In conclusion, the study shows that the RMA-Pearson method has better coverage of the coexpression landscape and the MAS5-Spearman is more accurate to find coexpression pairs. These results support the use of both methods in order to find a confident human coexpression network, since they do not find exactly the same expression signal and both provide important and complementary data allowing a progressive improvement of the significance and confidence of the coexpression set. Moreover, a better knowledge of the strength of each method is a discovery that complements previous comparative studies about RMA [7] and MAS5 [8].

### Effects of gene filtering

The original coexpression data used in **Figure 2** are obtained without any gene filtering, however for the analyses in **Figure 3** it was convenient to study the effect of gene filtering upon the accuracy and coverage of the methods. The evolution of the coverage did not show any significant change (data not shown). The evolution of the accuracy was studied by plotting the relative changes of the positive predictive values (PPV) of the coexpressing data with **r** (**Fig. 3** top graphs) and **N** (**Fig. 3** bottom graphs) for each method. In these graphs the **blue** circles correspond to non-filtered data and **red** circles to filtered data. This analysis indicates that for the case of RMA-Pearson method (**Fig. 3B**) a significant improvement was obtained with the gene filtered versus non-filtered. However, in the case of MAS5-Spearman there was not any relative improvement, as it can be seem in **Fig. 3A** both for **r** and **N**. This means that **r** and **N** are already very stringent in MAS5-Spearman dataset and the filter takes out approximately the same amount of estimated true positives and false positives within the data, and so it does not improve the coexpression accuracy (i.e. PPV). This observation, together with the fact that filtered data with the MAS5-Spearman method gives low coverage (as indicated above the total amount of positive pairs was only 2,198), brings us to the resolution of not using the filter for MAS5 dataset. By doing this, the MAS5-Spearman non-filtered dataset at **r** = 0.5 and **N** = 200 included 15,623 positive coexpression pairs; and this number was very similar to the 15,657 pairs found for RMA-Pearson filtered.

### Integration of correlation, cross-validation and PPV for datasets obtained with two balanced methods

**Figure 4:**
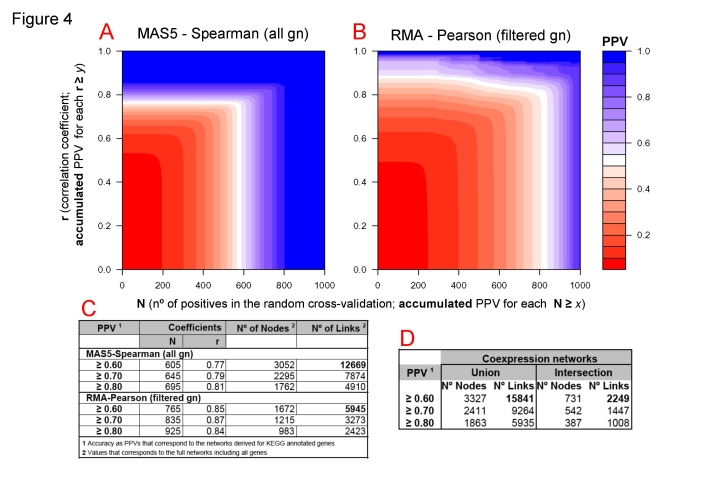
Coexpression networks obtained at different levels of accuracy. Color plots (A and B) that represent the Positive Predictive Value (PPV) calculated for each set of gene coexpression data for different values of correlation coefficient (r) and cross-validation coefficient (N). The PPV corresponds to accumulated values for N≥*x* and r≥*y*. Calculations are done for data derived from two methods: (A) MAS5-Spearman without gene filtering (all gn) and (B) RMA-Pearson with gene filtering (filtered gn). Table (C) shows the specific values of correlation and cross-validation for three coexpression datasets derived from each method at 3 specific PPVs: ≥0.60, ≥0.70 and ≥0.80. This table also shows the number of nodes and links included in each coexpression dataset. Table (D) shows the number of gene-nodes and interaction-links that are included in the combined coexpression networks at 3 specific PPVs.

Following the observations and arguments described above we proceed to integrate in “three-dimensions color plots” the data corresponding to the values of correlation (**r**), cross-validation (**N**) and PPV obtained with each method. The results are shown in **Figure 4**. The graphic considers all the calculated subsets of coexpression gene pairs and represents, for each one, the numerical relationship between the accumulated values of the estimated accuracy (**PPV**) corresponding to the correlation coefficients (**r** in *y* axis) and to the cross-validation coefficients (**N** in *x* axis). PPV ranges from 0.05 to 1.0 as indicated in the color scale of **Fig. 4**: **red** low and **blue** high. The graph are calculated for the data corresponding to two methods: MAS5-Spearman without gene filtering (all gn) (**Fig. 4A**) and RMA-Pearson with gene filtering (filtered gn) (**Fig. 4B**). As indicated above, in these conditions both methods include a similar number of coexpression pairs and so they are “balanced” with respect to the coverage.

The three-dimensions color plots allow to assess in a graphic way the level of confidence for a given coexpression data subset. We use them to select three data subsets derived from each method at three specific PPV values: ≥0.60, ≥0.70 and ≥0.80. The values of the correlation and cross-validation coefficients that correspond to these data subsets are indicated in the table enclosed as **Fig. 4C**. The figures show that the second method (RMA-Pearson) is more stringent, since the same given PPVs correspond to higher values of **N** and **r**. The size of the gene coexpression networks that correspond to the three selected accuracy values are also presented in **Fig. 4C**, including for each network the number of nodes (i.e. number of genes) and the number of links (i.e. number of coexpression pairwise relations). The selection and combination of these subsets at well defined and precise accuracy allows the identification of stable and confident human coexpression networks. This was done in the table enclosed as **Fig. 4D**, where the results of the union and the intersection of the datasets provided by the two methods at each PPV are presented. The union with accuracy ≥0.60 provides a full confident and cross-validated human gene coexpression network that includes 3327 genes and 15841 coexpression links. As indicated bellow, we have analyzed in detail a core transcriptomic network that corresponds to the intersection of both methods with accuracy ≥0.60 and includes 731 gene nodes and 2249 coexpression links.

### Biological significance of the coexpression datasets: house-keeping gene pairs and tissue-specific gene pairs

Once significant human gene coexpression datasets have been found and evaluated using statistical parameters, we started exploring the biological meaning and functional consistency of these datasets.

**Figure 5:**
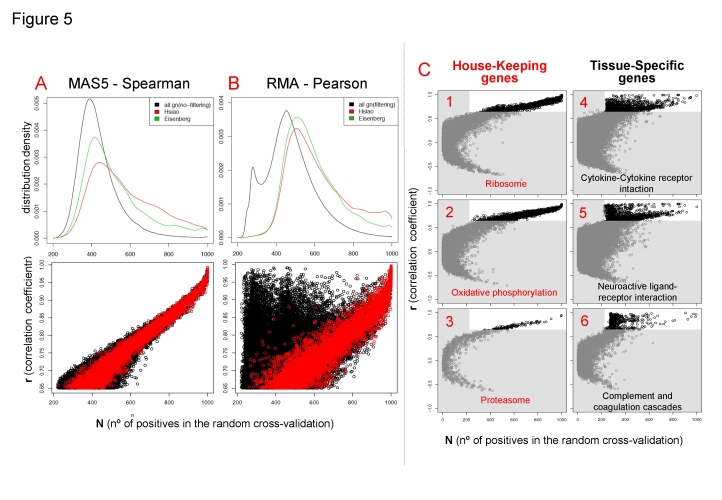
Coexpression of house-keeping and tissue-specific genes. Top panels A and B: Density distributions of coexpression data for N>220 corresponding to all gene pairs (in black), to Eisenberg's house-keeping gene pairs (in green) or to Hsiao's house-keeping gene pairs (in red). Bottom panels A and B: rN-plots with all data points of coexpression pairs with N>220 and r>0.65 for either all gene pairs (in black) or only Hsiao's house-keeping gene pairs (in red). In these panels (A) correspond to data from MAS5-Spearman method and (B) from RMA-Pearson method. Panels (C) 6 rN-plots that present the coexpression data obtained with the RMA-Pearson method corresponding to the human genes included in 6 different pathways: (1) ribosome (KEGG ID = hsa03010), (2) oxidative phosphorylation (hsa00190), (3) proteasome (hsa03050), (4) cytokine-cytokine receptor interaction (hsa04060), (5) neuroactive ligand-receptor interaction (hsa04080), and (6) complement and coagulation cascades (hsa04610).

In a first approach, we investigate the location of house-keeping gene pairs in the coexpression datasets, taking two different published compendiums of human house-keeping genes [16], [17]. *Hsiao et al.* identified 451 genes that are expressed in all 19 different human tissue types. *Eisenberg et al.* identified 575 human genes that show constitutive expression in all conditions tested in several publicly available databases. Mapping these genes in the general distribution of coexpression data shows that the ratio of house-keeping genes increases at high **N** and **r** coefficient values (**Fig. 5A,B**). The top panels in **Fig. 5A and B** present the density distributions of coexpression data for **N**>220 corresponding to all gene pairs (in **black**), to *Eisenberg's* house-keeping gene pairs (in **green**) or to *Hsiao's* house-keeping gene pairs (in **red**). Bottom panels in **Fig. 5A and B** show the same information including now all data points of coexpression pairs with **N**>220 and **r**>0.65 for either all gene pairs (in **black**) or only *Hsiao's* house-keeping gene pairs (in **red**). Panels **A** correspond to coexpression data obtained with method MAS5-Spearman and **B** to RMA-Pearson. The results reveal that house-keeping genes have a clear tendency to coexpress in many different tissues. This can be expected from the mere definition of house-keeping; however, since the result is obtained by mapping external datasets [16], [17] on our human gene coexpression data, it provides functional validity to our coexpression study. The analysis also reveals a clear difference between the data obtained with different methods. Meanwhile MAS5-Spearman method finds mainly house-keeping gene coexpression, the RMA-Pearson method finds many gene pairs that are not in the major house-keeping region, but rather they show high levels of **r** correlation with lower levels of **N** cross-validation (**N**>220 and **N**<600).

We further investigate this observation by selecting subsets of the coexpression data for genes included in specific KEGG pathways. Examples of this subsetting are presented in **Fig. 5C**, that includes 6 panels with the coexpression data obtained with the RMA-Pearson method for the human genes included in 6 different pathways: **(1)** ribosome (KEGG ID = hsa03010), **(2)** oxidative phosphorylation (hsa00190), **(3)** proteasome (hsa03050), **(4)** cytokine-cytokine receptor interaction (hsa04060), **(5)** neuroactive ligand-receptor interaction (hsa04080), and **(6)** complement and coagulation cascades (hsa04610). First three pathways can be considered as general constitutive, present in all tissues and cellular types. The other three pathways are tissue-specific, only present in some cell types, like: nervous system cells in the case of the neuroactive ligand-receptor interaction pathway or blood cells in the case of the complement and coagulation cascades pathway. These differences in functional specificity are reflected in the coexpression distributions: only the three panels on the right (**Fig. 5C 4,5,6**) present data points with high **r** values but relatively lower **N** values (220<**N**<600). In conclusion, this analysis reveals that such coexpression pairs correspond to genes expressed in specific cells or specific tissue types, and so they are tissue-specific genes.

### Comparison of human coexpression datasets: molecular machines and pathways consistently co-regulated

In a second approach, we investigate the functional assignment of the gene coexpression data following the strategy taken by *Stuart et al.* [5], who explored functional coverage on a coexpression network obtained for four organisms looking at the percentage of genes that are connected to at least one other gene in the same “functional category”. We proceed to the same percentage calculation using the KEGG pathways as “functional categories”. The analysis was done for the coexpression dataset derived from RMA-Pearson method with **r**>0.63 and **N**>500. The same functional analysis was also done using two other external human coexpression datasets previously published by *Lee et al.* [2] and *Griffith et al.* [6].

The results are presented in **Table 1**, that includes the ten-top pathways found with best percentage of genes coexpressing within the gene groups assigned to KEGG pathways for 3 different human coexpression datasets (this work, *Lee et al.* and *Griffith et al.*). This comparative analysis of functional coverage shows some interesting results: **(i)** All coexpression datasets find the most significant coexpression for 3 key molecular machines: ribosome, proteasome and oxidative phosphorylation. **(ii)** Genes involved in cell scaffolding and cell to cell interaction or anchoring are also found to coexpress quite often, as indicated by the presence of pathways like focal adhesion, extracellular matrix (ECM) interaction and cytoskeleton regulation. **(iii)** Genes involved in cell cycle pathway are also common to the three datasets, indicating that cells keep a tight regulation of the genes involved in essential living functions (maintenance, proliferation, survival). **(iv)** An important difference between our coexpression dataset and *Lee et al.* or *Griffith et al.* datasets is that this work only includes samples coming from normal non-pathological tissues, but the others include quite heterogeneous samples mixing normal and disease altered samples (for example, *Lee et al.* includes many human cancer samples). The inclusion of pathological samples can bias the results and this may be the reason of the appearance of “pathogenic infection pathways” in *Lee et al.* data. **(v)** Finally, the data obtained in this work also includes many coexpressing pairs involved in cell-cell communication like cytokine-receptor and ligand-receptor interactions.

**Table 1:** 

*This work (2008)*				
Pathway Name (KEGG ID number)	n° gn 1	gn coexp/gn 2	% gn coexp	mean r 3
**Proteasome (3050)**	31	28/28	**100.0%**	0.69
**Ribosome (3010)**	120	52/55	**94.5%**	0.75
**Oxidative phosphorylation (190)**	129	88/95	**92.6%**	0.73
Focal adhesion (4510)	194	154/168	**91.7%**	0.68
Antigen processing and presentation (4612)	86	71/78	**91.0%**	0.75
Glycan structures - degradation (1032)	30	20/22	**90.9%**	0.65
Neuroactive ligand-receptor interact. (4080)	299	227/255	**89.0%**	0.68
**Cell cycle (4110)**	114	90/102	**88.2%**	0.66
Regulation of actin cytoskeleton (4810)	208	141/161	**88.2%**	0.66
Cytokine-cytokine receptor interact. (4060)	256	196/223	**87.9%**	0.69

*Lee et al. (2004)*			
Pathway Name (KEGG ID number)	n° gn 1	gn coexp/gn 2	% gn coexp
**Ribosome (3010)**	120	43/44	**97.7%**
**Proteasome (3050)**	31	19/22	**86.4%**
**Oxidative phosphorylation (190)**	129	31/44	**70.5%**
**Cell cycle (4110)**	114	33/47	**70.2%**
ECM-receptor interaction (4512)	87	16/23	**69.6%**
Gap junction (4540)	92	9/13	**69.2%**
Pathogenic Escherichia coli infection (5130)	49	11/16	**68.8%**
Pathogenic Escherichia coli infection (5131)	49	11/16	**68.8%**
T cell receptor signaling pathway (4660)	93	15/22	**68.2%**
Metabolism of xenobiotics by cytP450 (980)	70	7/11	**63.6%**

*Griffith et al. (2005)*			
Pathway Name (KEGG ID number)	n° gn 1	gn coexp/gn 2	% gn coexp
**Ribosome (3010)**	120	36/38	**94.7%**
**Proteasome (3050)**	31	20/24	**83.3%**
**Oxidative phosphorylation (190)**	129	55/67	**82.1%**
Val, Leu and isoleucine degradation (280)	50	15/19	**78.9%**
ECM-receptor interaction (4512)	87	16/22	**72.7%**
**Cell cycle (4110)**	114	36/51	**70.6%**
Propanoate metabolism (640)	34	9/14	**64.3%**
Butanoate metabolism (650)	44	9/14	**64.3%**
Hematopoietic cell lineage (4640)	88	18/28	**64.3%**
beta-Alanine metabolism (410)	24	7/11	**63.6%**

1 n° gn = whole number of genes included in this KEGG pathway.

2 gn coexp/gn = genes that coexpress within the genes included for this pathway in the network.

3 mean value of the correlation factor (r) for the coexpressing gene pairs included in this pathway.

As a general conclusion of this analysis, we can say that KEGG pathways is revealed as a good database to investigate the biological functions of human genes, because it includes groups of genes that really work together in well defined biomolecular processes.

The comparative calculation of the coverage for the three human coexpression datasets included in **Table 1** indicates that the data obtained in this work present a higher level of functional coherence than previously published datasets [2], [6]. This comparison was also done taking coexpression networks of similar sizes (including in each case around 12,000 best coexpression relations) and calculating the statistical accuracy for all of them. The result presented in **Table 2** shows that the accuracy estimated as PPV was 0.61 for our dataset obtained with MAS5-Spearman, 0.56 for *Lee et al.* and 0.49 for *Griffith et al.* As a whole these numbers indicate that the human coexpression network derived from this work includes very consistent co-regulation of genes many times involved in common pathways.

**Table 2:** 

	Nodes 1	Links 2	TP 3	All 4	PPV 5
*This work (2008)*	3052	12669	729	1189	**0.613**
*Lee et al. (2004)*	1751	12187	1275	2265	**0.563**
*Griffith et al. (2005)*	2922	12686	1265	2588	**0.489**

1 N° of genes as nodes in the network (the values correspond to the full networks including all genes).

2 N° of coexpression links (the values correspond to the full networks including all links).

3 True Positives = gene-pairs that coexpress and are annotated to the same KEGG.

4 All the genes that coexpress and are annotated to KEGG.

5 Accuracy as PPVs that correspond to the networks derived for KEGG annotated genes.

### A high confidence human coexpression network reveals a map of ubiquitous biological functions

As far as we know, none of the previously published human coexpression networks [2], [5], [6] has a comprehensive calculation of the estimated statistical error in the datasets at different levels of coverage. However, following the analysis and data presented in **Figure 4** we can select coexpression datasets at specific thresholds of PPV accuracy. In order to gain in reliability, we can also combine the data obtained with 2 methods: MAS5-Spearman and RMA-Pearson. This was done taking the datasets of both methods with PPV≥0.60 (3052 and 1672 genes) to produce an intersect coexpression network that includes 731 genes and 2249 coexpression interactions (see **Fig. 4D**). We also restrict the network including only coexpressing groups including at least three genes. In this way, a high confidence core subset of 615 gene nodes and 2190 coexpression links was obtained.

**Figure 6:**
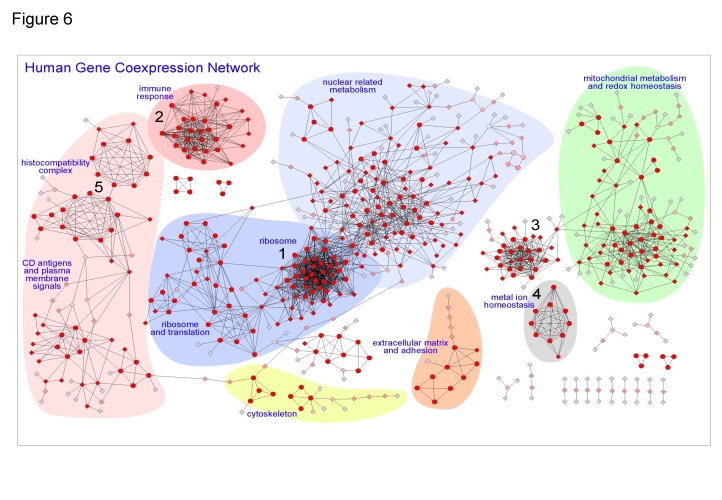
Human Gene Coexpression Network. Graphical view of the human gene coexpression network where the nodes correspond to genes and the edges to coexpression links. The network was produced as the intersection of two datasets (MAS5-Spearman and RMA-Pearson datasets with PPV≥0.60) to provide a confident coexpression network that includes 615 genes and 2190 pairwise coexpression interactions. The network includes only groups of coexpressing genes with at least three nodes. The most significant regions have been marked with background colors and labels describe main functions assigned. For each node the color (from red to grey) and shape (circles or diamonds) were obtained with MCODE algorithm. The circular nodes are the ones found with high cluster coefficient and the diamond nodes are the ones with lower cluster coefficient. The intensity of the red color in the nodes also indicates the degree of clustering, changing till pale grey for the most peripheral nodes that only have one link.

**Figure 6** presents a graphical view of this coexpression network where the nodes correspond to genes and the edges to coexpression. The network was produced introducing the coexpression dataset of 615 genes and 2190 pairwise interactions in *Cytoscape* (a bioinformatics software platform for visualizing molecular interaction networks, [18]. In the graphical view the most significant regions of this human gene coexpression network have been marked with background colors to enhance them as constellations within the coexpression landscape. Labels have been placed to each colored region to describe the main biological processes that are common to most of the genes in each region. The map shows that the larger sub-network corresponds to genes involved in nuclear activity and nuclear-driven metabolism (region in **blue**), with a side part (in dark **blue**) that includes most of the ribosomal proteins and proteins involved in ribosomal function. The second major constellation (region in **green**) includes many genes involved in mitochondrial metabolism and redox homeostasis (like genes of the COX family, the NDUF family and the UQCR family). The third main region (in **red**) corresponds to genes involved in the immune response, genes of the major histocompatibility complex (MHC), genes that produce the cell surface clusters of differentiation (CD) and genes that encode antigen-specific molecules. Finally some smaller regions include: genes involved in metal ion homeostasis (in **grey**); genes related to the extracellular matrix and cell adhesion (in **orange**); genes related to the cytoskeleton (in **yellow**).

As a whole the network is quite stringent but it is functionally very coherent. Moreover, coming from the intersection of two methods it will be expected to include mainly essential human genes. To prove if this network is enriched in house-keeping and essential genes we identified the nodes of the network that are included in the *Hsiao* human house-keeping gene set [16] and we also identified the nodes that correspond to genes that are orthologous to known essential yeast genes (taken from SGD database). In this way, we found that the two major constellations of the network, including mainly genes involved in nuclear related and mitochondrial related metabolism, show respectively 63% and 58% of genes assigned to be house-keeping. This result reveals that the coexpression network is enriched in essential genes.

In conclusion, the functional consistency observed in the constellations and regions defined by the coexpression network and the enrichment on house-keeping genes place the genes in a new integrative relational context that has strong biological coherence and, in many cases, can reveal essential or ubiquitous biological processes. The network also unravels new non-described human gene associations.

All the details about this coexpression network are provided in a supplementary file for *Cytoscape* (Supporting Information File S1: **S1_HumanCoexpNtw_615g_cys.zip**; that can be downloaded and used as a .*cys* file to be explored interactively using *Cytoscape*). This file also includes information about each node with GO and KEGG functional annotations.

### Analysis of the network with clustering algorithms

The network described above was analyzed using a graph theoretic clustering algorithm called MCODE [19] as indicated in **Materials and Methods**. The result of this analysis is presented in **Figure 6**, where the circular nodes are the ones with high “cluster coefficient” and the diamond nodes are the ones with lower “cluster coefficient”. The intensity of the **red** color of each node indicates the degree of clustering; changing up to pale **grey** for the most peripheral nodes (that only have one link). MCODE found 5 major gene coexpressing clusters marked with numbers in **Figure 6**: **(cluster 1)** corresponds to ribosomal genes, it includes 29 nodes and 366 links and many of the genes are RPL or RPS; **(cluster 2)** corresponds to immunoglobulins and immune response related genes (many belong to families IGH, IGK and IGL) and it includes 19 nodes and 151 interactions; **(cluster 3)** includes 19 nodes and 140 interactions and corresponds to an heterogeneous group of genes strongly clustered with no apparent common functional theme; **(cluster 4)** includes 9 nodes and 36 interactions and corresponds to genes related to metal ion homeostasis (several MT1 and MT2); and **(cluster 5)** corresponds to genes related to the major histocompatibility complex (MHC), it includes 17 nodes split in two clusters with 63 interactions, where most of the genes are HLA. There are other less dense clusters also found by MCODE that have lower score and significance for this algorithm.

We also applied another cluster algorithm for graphs called MCL [20] (see **Methods**). The analysis with MCL provided similar results to MCODE for the large clusters mentioned, although it splits the network in more clusters being the smaller ones more coherent in functional terms that the ones found by MCODE. For example, MCL algorithm finds another cluster form by 15 genes, with 7 assigned to RNA binding gene products, 3 to DNA binding gene products (all included in region **blue** in **Figure 6**), other 3 genes members of the gene family HNRP (heterogeneous nuclear ribonucleoproteins: HNRPA2B1, HNRPR, HNRPU) and 2 genes translation initiation factors (EIF3M, EIF4G2).

These results show that the gene clusters obtained with the graph algorithms from the coexpression network can help to understand the function of many human genes and the active relations between them. As expected, we find that stable and consistent coexpression clusters of genes are involved in specific functions, at cellular or systemic level. A complete analysis of all clusters is not possible in just one article but, as indicated above, the coexpression datasets of this study are open to new studies.

### Functional coherence of the coexpressing modules: finding coregulation and new biological assignments

To show some specific examples about the functional coherence of the gene coexpressing modules and the adequate correlation of the genes with common regulatory elements (i.e. transcription factors, TFs, and corresponding promoters) we analyzed three specific clusters or modules found in the core coexpression network.

**Figure 7:**
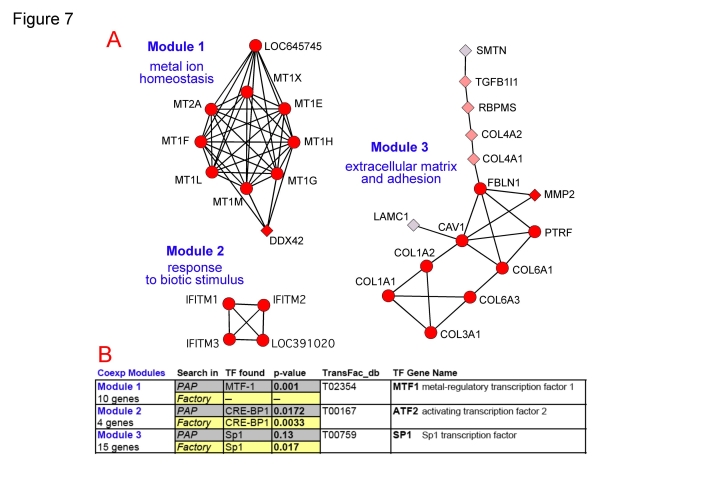
Coexpressed gene modules regulated by specific transcription factors. (A) Graphical enlarged view of three coexpressing modules selected from the network presented in Figure 6, indicating the name of each gene corresponding to each node and the functional labels: (Module 1) metal ion homeostasis; (Module 2) response to biotic stimulus; (Module 3) extracellular matrix and adhesion. (B) Table showing the results of the search for common transcription factors (TFs) most significantly associated to the genes included in each of the three modules described above. The search was done using the bioinformatic tools PAP and FactorY.

The first module includes 10 genes: 8 forming a full cross-related octogonal structure plus 2 nodes linked to them. The 8 genes are all metallothioneins: MT1E, MT1F, MT1G, MT1H, MT1L, MT1M, MT1X, MT2A. The other 2 genes are not well annotated: DDX42 (that encodes a member of the DEAD box protein family with unclear function) and LOC645745 (that has been recently and provisionally identified as a putative MT1, metallothionein 1 pseudogene 2). The coexpression of these two genes with a well defined and stable cluster of metallothioneins allows to infer that they will be genes also involved in metal ion homeostasis. This module can be seen in **Figure 7**.

A further analysis was done to find if these coexpressing genes have any common transcription factor (**TF**) that can act on the promoters and regulation regions of these genes. Two bioinformatic tools were used to find out **TFs** associated in a significant way to the coexpressing genes: PAP [21] and FactorY (see **Methods**). Using PAP we found that the 10 coexpressing genes of module 1 are regulated in common by the transcription factor MTF1 (found with p-value = 0.001). This result could be expected since MTF1 is a metal-regulatory transcription factor that induces expression of metallothioneins and other genes involved in metal homeostasis (such as zinc and copper). In any case, the association of MTF1 to module 1 provides strong coherence to the data, showing that this coexpression network is correlated with an underlying transcription regulatory entity.

The second module shown in **Figure 7** includes 4 genes: 3 correspond to interferon-induced transmembrane proteins (IFITM1, IFITM2, IFITM3) and the fourth is an unknown gene LOC391020 recently annotated by inference as similar to interferon-induced transmembrane protein 3. The coexpression of these four genes in a full related cluster gives support to the indication that all produce IFITM proteins. The analysis of transcription factors done with PAP and FactorY (**Figure 7B**) indicated that these 4 genes can be significantly correlated with the transcription factor CRE-BP1 (also called ATF2, activating transcription factor 2), that is a protein which binds to the cAMP-responsive element promoter (CRE, an octameric palindrome) and forms a homodimer or heterodimer with JUN. The deduction that IFITM genes can be coregulated by ATF2 makes biological sense because it has been observed that transcriptional activation of interferon related genes requires assembly of an enhanceosome containing the transcription factors ATF2 and JUN [22], [23].

Finally, the third module shown in **Figure 7** includes 15 genes: 6 encode for collagen proteins (COL1A1, COL1A2, COL3A1, COL4A1, COL4A2, COL6A1) that are fibrillar proteins found in most connective tissues, related to the extracellular matrix. Other proteins within this module are also related to cell adhesion and extracellular matrix, like: Fibulin 1 (FBLN1), a secreted glycoprotein that becomes incorporated into the fibrillar extracellular matrix; Laminin gamma 1 (LAMC1), another extracellular matrix glycoprotein which is part of the major noncollagenous constituent of basement membranes; and matrix metalloproteinase 2 (MMP2), that belongs to a family of proteins involved in the breakdown of extracellular matrix in normal physiological processes and in altered disease processes. In fact MMP2 gene encodes an enzyme which degrades type IV collagen. All these data indicate functional consistency and proximity for the genes included in this coexpression module. The analysis, using PAP and FactorY, of the regulatory promoters of this 15 genes shows a significant association with SP1 transcription factor, and recent experimental data have reported that in fact SP1 transcription factor is involved in the regulation of the collagen promoters [24]–[26].

The results presented for three coexpression modules can be extended to most of the clusters present in the network, and they indicate that the coexpression network can be correlated with an underlying regulatory network driven by specific transcription factors. This observation provides biological and functional coherence to the human gene pairwise coexpression network presented in this paper deduced from the analysis of normal-healthy human samples (whole tissues, glands or organs).

Finally, it is clear that a complete pairwise coexpression network of human genes will be only obtained using a comprehensive and systematic set of samples including all different human cell types. This achievement is at present quite far and difficult, since there are more than two hundred different cell types in the human body and that each cell type can be at different development or differentiation stages. Meanwhile, however, we think that the present study reports a reliable gene-gene coexpression network that includes very valuable information about many human genes, placing them in an integrated transcriptomic context. These coexpression networks selected at specific levels of confidence include a lot of information to better understand the complexity of the human expressing genome.

## Materials and Methods

### Sample selection: dataset of genome-wide expression microarrays from human normal whole tissues/glands/organs

The data used in this work corresponds to a set of human genome-wide expression microarrays hybridized with mRNA samples coming from different human tissues, glands or organs from healthy normal individuals. The complete list of tissues, glands and organs is: *adrenal gland*, *appendix*, *blood*, *bone marrow*, *brain*, *kidney*, *liver*, *lung*, *lymph node*, *muscle heart*, *ovary*, *pancreas*, *pituitary gland*, *prostate gland*, *salivary gland*, *skin*, *spinal cord*, *testis*, *thymus gland*, *thyroid gland*, *tongue*, *tonsil gland*, *trachea* and *uterus*. These 24 samples where selected from a larger set of 68 human samples (GEO GSE1133; Su et al. 2004) that also included some cell specific sources, like: lung bronchial epithelial cells HBEC, blood B-cells CD19 and T-cells CD4. The samples selection done was driven under the criteria of including mRNA samples from whole organs, glands or tissues covering the main parts of the human body and avoiding samples of very specific cell types within a tissue. This selection was validated performing global expression analyses of the samples, using a series of algorithms described bellow. The total mRNA from these 24 different samples came form a mix of 3 different individuals, that were: two men and one woman or one man and two women for the samples non sex-associated; three men for *testis* and *prostate* samples and three women for *ovary* and *uterus* samples. Moreover two biological replicates were used in each case, producing a total set of 48 microarrays. The microarrays used were high density oligonucleotide microarrays HGU133A GeneChips from *Affymetrix*, that include 22,283 probesets (corresponding to 13,068 human genes according to *Affymetrix* annotation).

### Genome-wide sample expression profiles and gene expression signatures

The global expression matrix including the genome-wide expression profiles of each sample and the expression signature of each gene-probeset was calculated and evaluated using a set of algorithms and methods in four consecutive steps: **(1^st^)** use of two different background correction, normalization and signal calculation methods: MAS5 [8], [27] and RMA [28]; **(2^nd^)** use of two distance measuring methods based in the global gene expression profile of each sample: first, distance based on Spearman correlation coefficient applied to MAS5 data; second, distance based on Pearson correlation coefficient applied to RMA data (both methods provided robust non-parametric distance distributions); **(3^rd^)** analysis by hierarchical clustering with complete linkage of the samples using the tool *hclust* from **R** (http://www.r-project.org/), taking as distance (**1−r**), where **r** is the correlation coefficient between sample expression profiles [29]; **(4^th^)** analysis by bootstrapping of the sample hierarchical trees to assay the stability of the associations, using the tool *pvclust* from **R**. The *pvclust* algorithm allows to assess the uncertainty in hierarchical cluster analysis via multiscale bootstrap resampling. This assessment is provided by two parameters: the *approximately unbiased p-value* (AU) and the *bootstrap probability value* (BP). The maximum and optimum values of AU and BP are 1 (or 100 in %).

### Gene pairs coexpression and cross-validation

As indicated above the global gene to gene (i.e. pair-wise) coexpression matrix was calculated using two different and independent methods: MAS5-Spearman and RMA-Pearson. Furtherly, cross-validation was used to discriminate stable and significant correlations. The cross-validation strategy applied was a 1000 times random selection of a 25% subset sampling (that are 12 samples, corresponding to 6 duplicates out of 24 duplicated samples) and calculation of the **r** correlation coefficient for each gene-probeset pair in such 1000 samplings. Only when the **r** correlation coefficient for a given time was higher than |0.70|, such was considered a positive event (positive cross-validation) and counted for the corresponding gene-probeset pair. In this way, for example, a given gene pair with **N** = 620 means that it gave 620 positive times out of the 1000 samplings. Therefore **N** can be considered a cross-validation coefficient or cross-validation factor (**N** = 620 is equivalent to 620/1000 = 0.62).

### Gene filtering method

In order to get rid of genes with low information content a combined filter based on between-sample variability and gene minimal signal was used. The filter leaves out only those gene probesets that fulfilled both of the two following conditions: **1^st^**.- Genes which have an expression difference or variability between samples (ΔExp^gi^_highest-lowest_) lower than the median of all the expression differences calculated for each gene (ΔExp^gi^_highest-lowest_<median ΔExp_highest-lowest_); **2^nd^**.- Genes which have a mean expression signal between samples (meanExp_samples_) lower than the median of all the expression signals calculated for each gene.

### Statistical estimation of accuracy and coverage of the coexpression datasets

The ***accuracy*** measured as “**Positive Predictive Value**” (**PPV**) in statistical terms is defined as the ratio TP/(TP+FP), where TP is the number of true positives and FP is the number of false positives [30], [31]. This parameter is related to “error type I”, and it is the inverse to the ratio of “false positives” (i.e. FP/(TP+FP), percentage of false positives within all the positives). The ***coverage*** (sometimes also named recall) can be measured as the proportion of true positives that remain in a given subset selected, with respect to an initial reference set of positives. We consider that both the accuracy and coverage are critical statistical parameters to evaluate the error and validity of a method. They are directly related to ***specificity*** = TN/(TN+FP), −where (TN+FP) are all the “false”−, and ***sensitivity*** = TP/(TP+FN) −where (TP+FN) are all the “true”− [30], though these can only be applied when the real true and real false data of a test are known; while the accuracy defined as “positive predictive value” and the defined coverage can be applied when it is only possible to know or estimate the “positive data”.

Therefore, in this study if the true data are not known (i.e. if we do not know *a priori* which are true gene coexpressing pairs) a proper calculation of the sensitivity and specificity is not possible. This is the most common situation in many biological and biomolecular studies where many of the true occurring relations between molecules are not yet known. Therefore, we need to design a way to at least estimate the percentage or ratio of “true positives” of the method, and so estimate the accuracy and coverage. These parameters will provide a good indication of how valuable is the method that we have applied to find human coexpressing gene pairs. The estimation was done considering the idea that genes that work together in the same biological pathway are much more likely to coexpress than genes that are not involved in a common biological reaction or pathway. This biomolecular axioma in our case was tested annotating all the genes of the microarrays to the KEGG pathway database (www.genome.jp/kegg/), that is one of the most complete and expert curated repository of human genes involved in biological reactions or pathways [32]. Therefore, selecting only the subset of the genes annotated to KEGGs, a gene coexpression pair was considered a “true positive” when both genes of the pair were included in a common KEGG human pathway. This strategy allows to calculate the statistical parameters ***accuracy*** and ***coverage*** defined above, and therefore to explore how the values of the **r** and **N** coefficients change such parameters.

### Analytic algorithms to find groups and modules in the coexpression networks

The gene to gene coexpression networks obtained were analyzed using a graph theoretic clustering algorithm called MCODE (Molecular Complex Detection) [19] that allows to detect densely connected regions in large interaction networks which may represent molecular associations. This algorithm follows a vertex weighting by local neighbourhood density and outward traversal from locally dense seed nodes to isolate the dense regions. Furthermore, the networks were also analyzed using another cluster algorithm for graphs called MCL (Markov Cluster algorithm, http://micans.org/mcl/) [20] that finds cluster structure in graphs by a mathematical bootstrapping procedure. MCL has been shown very robust to find relevant modules in protein interaction networks [33].

### Mapping transcription factors associated to gene coexpressing modules

Two bioinformatic tools were used to find out transcription factors that can be associated in a significant way to groups or modules of coexpressing genes: Promoter Analysis Pipeline (PAP) and Transcription Factor Enrichment Analysis (FactorY).

PAP is based in a systematic, statistical model of mammalian transcriptional regulatory sequence analysis and it is suitable for the identification of the potential transcriptional regulators of co-expressed genes and the identification of the potential regulatory targets of transcription factors. A typical PAP analysis includes input of a co-expressed gene cluster, identification of several high scoring transcription factors and visualization of the predicted transcription factor binding sites [21]. The bioinformatic tool is at: http://bioinformatics.wustl.edu/webTools/portalModule/PromoterSearch.do.

FactorY is another bioinformatic tool that explores the 1000 bp upstream sequence signature of co-expressed genes to find homology with transcription factor binding sites (TFBs) based on JASPAR and TRANSFAC databases. The tool calculates the significant enrichment in known given TFBs for a group of genes and it was used at the web site: http://www.garban.org/factory/.

## Supporting Information

File S1 Human Gene Coexpression Network. Network that corresponds to the core with the most confident human gene pairwise coexpression data and includes 615 gene-nodes and 2190 coexpression-links. This network is provided in Cytoscape format (.cys file compressed as .zip) with full annotations about the genes. The file to be run in Cytoscape should have .cys extension: S1_HumanCoexpNtw_615g.cys
(0.30 MB ZIP)

## References

[1] van Noort V, Snel B, Huynen MA (2004). The yeast coexpression network has a small-world, scale-free architecture and can be explained by a simple model.. EMBO Rep.

[2] Lee HK, Hsu AK, Sajdak J, Qin J, Pavlidis P (2004). Coexpression analysis of human genes across many microarray data sets.. Genome Res.

[3] Tirosh I, Weinberger A, Carmi M, Barkai N (2006). A genetic signature of interspecies variations in gene expression.. Nat Genet.

[4] Magwene PM, Kim J (2004). Estimating genomic coexpression networks using first-order conditional independence.. Genome Biol.

[5] Stuart JM, Segal E, Koller D, Kim SK (2003). A gene-coexpression network for global discovery of conserved genetic modules.. Science.

[6] Griffith OL, Pleasance ED, Fulton DL, Oveisi M, Ester M (2005). Assessment and integration of publicly available SAGE, cDNA microarray, and oligonucleotide microarray expression data for global coexpression analyses.. Genomics.

[7] Bolstad BM, Irizarry RA, Astrand M, Speed TP (2003). A comparison of normalization methods for high density oligonucleotide array data based on variance and bias.. Bioinformatics.

[8] Lim WK, Wang K, Lefebvre C, Califano A (2007). Comparative analysis of microarray normalization procedures: effects on reverse engineering gene networks.. Bioinformatics.

[9] Suzuki R, Shimodaira H (2006). Pvclust: an R package for assessing the uncertainty in hierarchical clustering.. Bioinformatics.

[10] Wang Y, Miao ZH, Pommier Y, Kawasaki ES, Player A (2007). Characterization of mismatch and high-signal intensity probes associated with Affymetrix genechips.. Bioinformatics.

[11] Barnes M, Freudenberg J, Thompson S, Aronow B, Pavlidis P (2005). Experimental comparison and cross-validation of the Affymetrix and Illumina gene expression analysis platforms.. Nucleic Acids Res.

[12] Dallas PB, Gottardo NG, Firth MJ, Beesley AH, Hoffmann K (2005). Gene expression levels assessed by oligonucleotide microarray analysis and quantitative real-time RT-PCR – how well do they correlate?. BMC Genomics.

[13] Choi JK, Yu U, Yoo OJ, Kim S (2005). Differential coexpression analysis using microarray data and its application to human cancer.. Bioinformatics.

[14] Prieto C, Rivas MJ, Sanchez JM, Lopez-Fidalgo J, De Las Rivas J (2006). Algorithm to find gene expression profiles of deregulation and identify families of disease-altered genes.. Bioinformatics.

[15] Calza S, Raffelsberger W, Ploner A, Sahel J, Leveillard T (2007). Filtering genes to improve sensitivity in oligonucleotide microarray data analysis.. Nucleic Acids Res.

[16] Hsiao LL, Dangond F, Yoshida T, Hong R, Jensen RV (2001). A compendium of gene expression in normal human tissues.. Physiol Genomics.

[17] Eisenberg E, Levanon EY (2003). Human housekeeping genes are compact.. Trends Genet.

[18] Shannon P, Markiel A, Ozier O, Baliga NS, Wang JT (2003). Cytoscape: a software environment for integrated models of biomolecular interaction networks.. Genome Res.

[19] Bader GD, Hogue CW (2003). An automated method for finding molecular complexes in large protein interaction networks.. BMC Bioinformatics.

[20] Enright AJ, Van Dongen S, Ouzounis CA (2002). An efficient algorithm for large-scale detection of protein families.. Nucleic Acids Res.

[21] Chang LW, Fontaine BR, Stormo GD, Nagarajan R (2007). PAP: a comprehensive workbench for mammalian transcriptional regulatory sequence analysis.. Nucleic Acids Res.

[22] Falvo JV, Parekh BS, Lin CH, Fraenkel E, Maniatis T (2000). Assembly of a functional beta interferon enhanceosome is dependent on ATF-2-c-jun heterodimer orientation.. Mol Cell Biol.

[23] Panne D, Maniatis T, Harrison SC (2004). Crystal structure of ATF-2/c-Jun and IRF-3 bound to the interferon-beta enhancer.. Embo J.

[24] Kypriotou M, Beauchef G, Chadjichristos C, Widom R, Renard E (2007). Human collagen Krox up-regulates type I collagen expression in normal and scleroderma fibroblasts through interaction with Sp1 and Sp3 transcription factors.. J Biol Chem.

[25] Magee C, Nurminskaya M, Faverman L, Galera P, Linsenmayer TF (2005). SP3/SP1 transcription activity regulates specific expression of collagen type X in hypertrophic chondrocytes.. J Biol Chem.

[26] Poree B, Kypriotou M, Chadjichristos C, Beauchef G, Renard E (2008). Interleukin-6 (IL-6) and/or Soluble IL-6 Receptor Down-regulation of Human Type II Collagen Gene Expression in Articular Chondrocytes Requires a Decrease of Sp1{middle dot}Sp3 Ratio and of the Binding Activity of Both Factors to the COL2A1 Promoter.. J Biol Chem.

[27] Liu WM, Mei R, Di X, Ryder TB, Hubbell E (2002). Analysis of high density expression microarrays with signed-rank call algorithms.. Bioinformatics.

[28] Irizarry RA, Bolstad BM, Collin F, Cope LM, Hobbs B (2003). Summaries of Affymetrix GeneChip probe level data.. Nucleic Acids Res.

[29] Murtagh F (1985). Multidimensional Clustering Algorithms. COMPSTAT Lectures.

[30] Loong TW (2003). Understanding sensitivity and specificity with the right side of the brain.. Bmj.

[31] Suojanen JN (1999). False false positive rates.. N Engl J Med.

[32] Aoki-Kinoshita KF, Kanehisa M (2007). Gene annotation and pathway mapping in KEGG.. Methods Mol Biol.

[33] Brohee S, van Helden J (2006). Evaluation of clustering algorithms for protein-protein interaction networks.. BMC Bioinformatics.

